# Associations between factors within the home setting and screen time among children aged 0–5 years: a cross-sectional study

**DOI:** 10.1186/1471-2458-12-539

**Published:** 2012-07-23

**Authors:** Valerie Carson, Ian Janssen

**Affiliations:** 1 School of Kinesiology and Health Studies, Queen’s University, Kingston, ON, Canada; 2 Department of Community Health and Epidemiology, Queen’s University, Kingston, ON, Canada

**Keywords:** Child, Pre-school, Television, Parents

## Abstract

**Background:**

Excessive engagement in screen time has several immediate and long-term health implications among pre-school children. However, little is known about the factors that influence screen time in this age group. Therefore, the purpose of this study was to use the Ecologic Model of Sedentary Behavior as a guide to examine associations between intrapersonal, interpersonal, and physical environment factors within the home setting and screen time among pre-school children.

**Methods:**

Participants were 746 pre-school children (≤ 5 years old) from the Kingston, Ontario, Canada area. From May to September, 2011, parents completed a questionnaire regarding several intrapersonal (child demographics), interpersonal (family demographics, parental cognitions, parental behavior), and physical environment (television, computer, or video games in the bedroom) factors within the home setting. Parents also reported the average amount of time per day their child spent watching television and playing video/computer games. Associations were examined using linear and logistic regression models.

**Results:**

Most participants (93.7%) watched television and 37.9% played video/computer games. Several intrapersonal, interpersonal, and physical environment factors within the home setting were associated with screen time. More specifically, age, parental attitudes, parental barriers, parental descriptive norms, parental screen time, and having a television in the bedroom were positive predictors of screen time; whereas, parental education, parental income, and parental self-efficacy were negative predictors of screen time in the linear regression analysis. Collectively these variables explained 64.2% of the variance in screen time. Parental cognitive factors (self-efficacy, attitudes, barriers, descriptive norms) at the interpersonal level explained a large portion (37.9%) of this variance.

**Conclusions:**

A large proportion of screen time in pre-school children was explained by factors within the home setting. Parental cognitive factors at the interpersonal level were of particular relevance. These findings suggest that interventions aiming to foster appropriate screen time habits in pre-school children may be most effective if they target parents for behavioral change.

## Background

Sedentary behavior is an emerging area of study. A major source of sedentary behavior in young people is screen time, which refers to time spent watching television or movies, playing video games, and using computers [1]. Pediatric organizations recommend no more than 1–2 hours of daily screen time for children aged 2–5 years and discourage screen time for children younger than the of age 2 [2-4]; unfortunately, many children are not meeting these recommendations. For example, an American study reported that 90% of children had already begun watching television by age 2 [5] and a Canadian survey reported that ~25% of children aged 2–5 watch >2 hours of television daily [6]. These statistics are concerning as excessive screen time in young children is associated with obesity [7], aggressive behavior [8], and may negatively impact attention span, language development, and cognitive development [9]. Furthermore, screen time habits formed at an early age may track overtime [10] and predict negative health outcomes later in life [11]. Thus, fostering appropriate screen time habits in pre-school aged children (i.e., 0–5 years old) may have important implications for health and wellness throughout life [6].

In order to develop evidence-based interventions to foster appropriate screen time habits in pre-school children the factors that influence the behavior need to be understood [12]. Behavior theories and models provide a systematic framework for examining the factors that influence behavior [13]. Owen and colleagues recently developed an Ecologic Model of Sedentary Behavior [14]. Consistent with other ecological models [15], this model postulates that sedentary behavior is influenced by factors at multiple levels including intrapersonal, interpersonal, physical environment, and policy factors [14]. However, according to this model, these factors may differ depending on the setting where the sedentary behavior occurs [14]. For example, the factors that influence sedentary behavior in pre-school children would likely be different in a home versus daycare setting. Therefore, they recommend setting-specific examinations of the multi-level factors that influence sedentary behavior. Since most or all screen time occurs at home for pre-school children [16], the home setting is important when examining the factors that influence screen time in this age group.

Several intrapersonal (e.g., age), interpersonal (e.g., parental television watching, parental rules), and physical environment (e.g., television in child’s bedroom) factors within the home setting are related to screen time among school-aged children and youth [17,18]. Less is known about these relationships in pre-school children. Since the early years is a distinct developmental period, the factors that influence screen time among school-aged children and youth may not be relevant for this age group. Two recent reviews that examined the factors that influence screen time use in young children (≤7 years old) and pre-school children (3–5 years old) reported that the findings from the existing literature are inconclusive [19,20]. In fact, the only consistently reported factor across both reviews was parental television viewing rules [19,20]. Many other potentially important factors such as attitudes [5], barriers [21], perceived norms [5], and self-efficacy [22] of parents have been examined too infrequently to draw conclusions [20]. Furthermore, most published studies have been atheoretical in nature and have examined factors based on associations observed for moderate-to vigorous-intensity physical activity (MVPA); even though, MVPA is distinct from sedentary behavior [23]. Therefore, the purpose of this study was to use the Ecologic Model of Sedentary Behavior as a guide to examine the influence of several intrapersonal, interpersonal, and physical environment factors within the home setting on screen time among children ≤5 years old.

## Methods

### Participants

This study is based on the Healthy Living Habits in Pre-school Children project. Data were collected between May and September 2011 on children ≤5 years old from the Kingston, Frontenac, Lennox, and Addington Health Region in Ontario, Canada. Parents with children ≤5 years old were recruited from two sources: licensed child care centers (46 of 60 participated) and public health/community programs (14 of 16 participated). Eligible parents received a package containing a questionnaire that had been pilot tested. The questionnaire was to be completed by the parent most familiar with the child and if the parent had more than one child ≤5 years old, it was to be completed for the child with the birth date closest to January 1^st^ or the oldest twin. The questionnaire required ~15 minutes to complete and was returned (or completed on the internet) in a pre-stamped and pre-addressed envelope. Approximately 37% of parents who received the packages completed the questionnaire (35% for child care centers and 40% for public health/community programs) resulting in a total sample of 800. Participants were excluded with missing child’s birth date or gender, leaving 746. Ethics approval was obtained from the Queen’s University General Research Ethics Board. Consent was obtained from participating child care centers, public health/community programs, and parents.

### Independent variables

#### Intrapersonal factors

##### Child demographic information

Parents reported their child’s gender and their month and year of birth.

#### Interpersonal factors

##### Family demographic information

Information was obtained on the presence of siblings in the household (0 = no; 1 = yes), family structure (0 = two-parent; 1 = single-parent homes), education (six categories ranging from ‘no schooling’ to ‘graduate university’), and annual household income (five categories ranging from ‘<$25,000’ to ‘≥$100,000’). These questions were adopted from Statistics Canada’s National Longitudinal Survey of Children and Youth [24].

##### Self-efficacy

Parents’ confidence in reducing or eliminating their child’s screen time was assessed by one item: “*How confident are you that you could say no to your child’s request to participate in screen time (TV/computer/video games) activities?*” It was rated on a 4-point scale ranging from ‘not at all confident’ to ‘very confident’. This item was developed using qualitative data collection and existing measures of personal self-efficacy [22]. It has demonstrated good reliability in mothers of 1-year and 5-year-old children [22].

##### Attitudes

Parents’ attitudes regarding their child’s screen time were assessed with eight previously developed [5] items: (1) “*It is good for his/her brain*”, (2) “It *is something my child finds very enjoyable*”, (3) “*It gives me the opportunity to get things done on my own*”, (4) “*It allows me to cope from a busy day at work and/or looking after multiple children*”, (5) “*My child needs/wants time to relax*”, (6) “*It is family time, bonding time, or quality time*”, (7) “*It grabs hold of my child’s attention*”, and (8) “*It teaches my child to get along with others*”. All items were rated on a 4-point scale ranging from ‘strongly disagree’ to ‘strongly agree’. Parents whose children did not engage in screen time (n = 47) did not respond to these items. To avoid excluding these participants, they were assigned the best response option (strongly disagree). Responses from all eight items were averaged to create an overall attitude score; higher scores reflected more positive attitudes. The internal consistency for the attitude items in this study was α = 0.84.

##### Barriers

Parents’ barriers to reduce their child’s screen time were assessed by six items: (1) “*There is pressure from society to purchase and use media-related equipment”,* (2) “*My neighborhood is not safe for my child to play outdoors*”, (3) “*Poor weather limit my child’s opportunities to go outside*”, (4) “*I need a coping-tool to meet the demands of a busy day at work or raising multiple children*”, (5) “*I need time to do household chores”,* and *(6) “My child really enjoys screen time activities”*. All items were rated on a 4-point scale ranging from ‘strongly disagree’ to ‘strongly agree’. Parents whose children did not engage in screen time were assigned the best response option (strongly disagree) for all items. The responses from the six items were averaged to create an overall barriers score; higher scores reflected more barriers. These items were developed from qualitative work on screen time in pre-school children [21,25]. The internal consistency for the barrier items in this study was α = 0.79.

##### Descriptive norms

Parents’ perception of typical screen time in children was assessed by one item: “*What do you feel is the maximum amount of time your child should spend participating in screen time (TV/computer/videogames) activities per day?*” It was rated on a 6-point scale ranging from ‘0 mins/day’ to ‘≥3 hours/day’. This item was modified from an existing measure of descriptive norms [5].

##### Parent’s screen time

The typical weekly time parents spent watching television/videos/DVDs, using a computer (not for work/school), and playing video games was assessed with the following 3 items: “*In a typical week in the past 3 months, how much time did you usually spend watching television, videos, or DVDs?*”, “*In a typical week in the past 3 months, how much time did you usually spend on a computer, including playing computer games and using the internet? (Do not include time spent at work or at school)*”, and “*In a typical week in the past 3 months, how much time did you usually spend playing video games (*e.g.*, Playstation, Wii, XBOX)?”*All items were rated on an 8-point scale ranging from ‘none’ to ‘>20 hours’. Screen time was determined by adding television, computer, and video game use. These questions were adopted from Statistics Canada’s Canadian Community Health Survey [26].

#### Physical environment factors

##### Television, computer, or video games in child’s bedroom

Parents reported whether their child had a television/portable DVD player (0 = no; 1 = yes), a computer (including a learning laptop, laptop, netbook, iPad; 0 = no; 1 = yes) or video game console (e.g., leapfrog Leapster, Playstation, Wii, XBOX; 0 = no; 1 = yes) in their bedroom.

### Dependent variables

#### Children’s screen time

The amount of time children spent watching television and playing video/computer games was assessed with two items: (1) “*On average, how much time per day does your child watch television, videos, or DVDs?*”, and (2) “*On average, how much time per day does your child play video/computer games?*” Both items were rated on a 7-point scale for weekday and weekend use ranging from ‘none’ to ‘≥3 hours/day’. Weighted means of weekday and weekend use were calculated and screen time was determined by adding television and video/computer games use. These questions were adopted from Statistic Canada’s NLSCY [24]. Parental report is commonly used in the literature to assess young children’s television viewing [5]. A previous validation study reported that a brief parental questionnaire used to measure children’s television viewing, similar to that used in the present study, was significantly correlated (r = 0.60) with children’s television viewing measured by a parental diary [27].

### Data analysis

Analyses were completed using SAS version 9.2 (SAS Institute Inc., Cary, NC). Screen time was positively skewed so it was square root transformed for correlation and regression analyses. Error estimates and 95% confidence intervals were adjusted for in the regression analyses using the SURVEYREG or SURVEYLOGISTIC procedures to account for clustering by child care center and public health/community program. A multiple imputation procedure with 5 imputations was performed for missing independent variables (excluding child’s age and gender) [28]. Of the total sample, 74% were not missing any independent variables, 19% were missing a single independent variable, and only 7% were missing 2 or more independent variables.

Descriptive statistics were calculated. Associations between variables were determined using Pearson correlations. A sequential linear regression model with 5 steps was used to predict children’s screen time. After step 1, each step adjusted for the variables included in the previous step(s). Only independent variables significantly (*P* ≤ 0.05) correlated with screen time were entered in the model. Based on the Ecologic Model of Sedentary Behavior [14], child demographic information (age, gender) were entered at step 1, representing intrapersonal level factors. Interpersonal level factors were entered in steps 2 (family demographics), 3 (parental cognitive factors), and 4 (parental behavioral factors). The physical environment factors (television, computer, video games in the bedroom) were added in step 5. The adjusted R-square was determined at each step. After step 5, age and gender interactions were explored. A sample size of *n* = 746 was deemed sufficient to detect an medium effect (i.e., *r* =0.30) at an alpha level of 0.05 and 80% power [29].

Additional analyses were conducting using a multiple logistic regression model to examine the associations between the independent variables and high screen time (top quartile or ≥107 mins/day). A manual backward elimination process with a *P* = 0.10 cut-off was used to determine the most parsimonious model. Finally, sensitivity analyses were conducted to explore whether the results were influenced by using imputed versus best response values for parental attitudes and barriers among the 47 participants who did not engage in screen time.

## Results

Participant characteristics are in Table 1. Just over half (53.5%) of the children were male and the average age was 41 (16 SD) months or 3 (1 SD) years. Most children (93.7%) watched television and 37.9% played video/computer games. The average min/day was 66.6 (48.0 SD) for television, 8.2 (18.6 SD) for video/computer games, and 74.8 (56.9 SD) for total screen time. For screen time, 13.6 engaged in >2 hour/day and 43.5% engaged in >1 hour/day.

**Table 1:** Participant characteristics (%)

	**Total (N = 746)**
Child’s Gender	
Male	53.5
Female	46.5
Parent’s Gender	
Male	7.0
Female	92.0
Other	1.0
Child’s Age	
< 1 years old	4.3
1-3 years old	34.7
4-5 years old	61.0
Sibling(s)	
No	37.7
Yes	62.3
Family Structure	
Two parent home	82.6
Single parent home	17.4
Parental Education	
Elementary (Grades 1–8)	1.5
High School (Grades 9–12)	14.1
Community/Technical College	35.3
University	27.9
Graduate University	21.2
Annual Household Income	
Less than $25,000	13.7
$25,000 to $50,000	15.3
$50,001 to $75,000	14.9
$75,001 to $100,000	17.6
More than $100,000	33.4
Not reported	5.1
Child Watches Television	
No	6.3
Yes	93.7
Child Plays Video/Computer Games	
No	62.1
Yes	37.9
Screen Time	
>1 hour/day	43.5
>2 hour/day	13.6

All of the independent variables, with the exception of the child’s gender and having a computer in the bedroom, were correlated with screen time (Table 2). Age, parental attitudes, parental barriers, parental descriptive norms, parental screen time, having a television in the bedroom, and having video games in the bedroom were positive correlates of screen time. Parental education, parental income, and parental self-efficacy were negative correlates of screen time. The strongest correlations were for attitudes (*r* = 0.62), descriptive norms (*r* = 0.58), and barriers (*r* = 0.54). None of the correlations between the independent variables were above *r* = 0.52 (data not shown).

**Table 2:** **Bivariate Pearson correlations (*r*) between screen time and the independent variables**

**Independent Variables**	**Screen Time (N = 746)**
Intrapersonal Factors	
Age	0.41**
Gender	−0.06
Interpersonal Factors	
Family Demographics	
Siblings	0.08*
Education	−0.22**
Income	−0.18**
Family Structure	0.08*
Parental Cognitions	
Self-efficacy	−0.26**
Attitudes	0.62**
Barriers	0.54**
Descriptive Norms	0.58**
Parental Behavior	
Parental Screen Time	0.29**
Physical Environment Factors	
Television in Bedroom	0.29**
Computer in Bedroom	0.06
Video Games in Bedroom	0.15**

Gender: 0 = boys, 1 = girls; siblings: 0 = no, 1 = yes; family structure: 0 = two-parent family, 1 = one-parent family; television in bedroom: 0 = no, 1 = yes; computer in bedroom: 0 = no, 1 = yes; video games in bedroom: 0 = no, 1 = yes * P < 0.01; ** P < 0.001.

The results of the sequential linear regression in the total sample (Table 3) indicated that 64.2% of the variance in children’s screen time was explained by the independent variables. Approximately 38% of the unique variance was explained by the parental cognitive variables at the interpersonal level (step 3), where parental attitudes, parental barriers, and parental descriptive norms were positive predictors and parental self-efficacy was a negative predictor of screen time. Most of the remaining variance was explained by age at the intrapersonal level (16.6% at step 1) and family demographics at the interpersonal level (8.1% at step 2). Age was a positive predictor of screen time; whereas, parental education and parental income were both negative predictors. While parental screen time at the interpersonal level (step 4) and television in the bedroom at the physical environment level (step 5) were both positive predictors of screen time, they explained little additional variance.

**Table 3:** Sequential linear regression models predicting screen time

	**0- to 5-year-olds (N = 746)**			**0- to 3-year-olds (N = 291)**			**4- to 5-year-olds (N = 455)**		
	**β**	**95% CI**	**R**^**2**^**(Unique)**	**β**	**95% CI**	**R**^**2**^**(Unique)**	**β**	**95% CI**	**R**^**2**^**(Unique)**
Intrapersonal Factors (Step 1)									
Age	0.09*	0.07, 0.11	0.166	0.22*	0.17, 0.28	0.251	0.03	<−0.01, 0.05	0.005
Interpersonal Factors									
Family Demographics (Step 2)			0.081			0.076			.114
Siblings	0.07	−0.50, 0.65		−0.36	−1.21, 0.49		0.16	−0.46, 0.77	
Education	−0.60*	−0.89, -0.31		−0.65*	−1.13, -0.17		−0.54*	−0.87, -0.22	
Income	−0.49*	−0.70, -0.27		−0.51*	−0.85, -0.18		−0.55*	−0.79, -0.30	
Family Structure	−0.69	−1.51, 0.13		−1.11	−2.15, 0.23		−0.54	−1.34, 0.26	
Parental Cognitions (Step 3)			0.379			0.416			0.335
Self-efficacy	−0.38*	−0.64, -0.11		−0.24	−0.70, 0.21		−0.52*	−0.87, -0.16	
Attitudes	1.68*	1.19, 2.17		2.20*	1.52, 2.59		0.87*	0.15, 1.60	
Barriers	0.99*	0.57, 1.42		0.80*	0.21, 1.39		0.90*	0.37, 1.42	
Descriptive Norms	1.16*	0.97, 1.35		1.08*	0.75, 1.40		1.19*	0.99, 1.40	
Parental Behavior (Step 4)			0.007			<0.001			0.029
Parental Screen Time	0.13*	0.07, 0.19		0.07	−0.04, 0.17		0.21*	0.13, 0.29	
			0.009						
Television in Bedroom	1.02*	0.45, 1.58		0.83	−0.2, 1.87	0.008	0.98*	0.27, 1.68	0.018
Video Games in bedroom	0.82	−0.16, 1.80		5.14*	3.79, 6.51		0.85	−0.11, 1.81	

The screen time dependent variable was square root transformed.

B = Unstandardized beta coefficient; 95% CI = 95% confidence interval; R^2^ = adjusted R-square; * *P* ≤ 0.05.

Total model *R*^2^ = 0.642 for 0- to 5-year-olds, 0.751 for 0- to 3-year-olds, and 0.501 for 4- to 5-year-olds.

There were no significant gender interactions with any of the independent variables and subgroup analyses revealed that R-square values from the sequential linear regression were similar in boys (R^2^ = 0.63) and girls (R^2^ = 0.66). Significant age interactions were observed with education and attitudes (*P* < 0.05). Subgroup analyses revealed that education and attitudes were stronger predictors of screen time in 0- to 3-year olds compared to 4- to 5-year-olds (Table 3). Meaningful differences in R-square values between 0- to 3-year-olds (R^2^ = 0.75) and 4- to 5-year-olds (R^2^ = 0.50) was also observed. Age differences existed at all 5 steps, with the largest differences occurring at steps 1 and 3. In step 1, age explained 25.1% of the variance in 0- to 3-year-olds compared to 0.5% in 4- to 5-year-olds. In step 3, parental cognitive variables explained a further 41.6% in 0- to 3-year-olds compared to 33.5% in 4- to 5-year-olds.

The results of the logistic regression analysis (Table 4) revealed that the significant predictors of high screen time in the total sample were similar to that of the continuous screen time variable (Table 3). Specifically, age, parental attitudes, parental descriptive norms, parental screen time, having a television in the bedroom, and having video games in the bedroom were positive predictors of high screen time; whereas, gender (being female), parental income, and parental self-efficacy were negative predictors of high screen time. Based on epidemiological standards, all of these odds ratios were modest in effect size, with the exception of parental income and having a television in the bedroom, which were weaker effects [30].

**Table 4:** Multiple logistic regression models predicting high (top quartile) screen time

	**Total Screen Time**	
	**OR**	**(95% CI)**
Intrapersonal Factors		
Age (per year)	1.81*	1.42, 2.30
Gender		
Male	1.00	
Female	0.52*	0.34, 0.80
Interpersonal Factors		
Family Demographics		
Income (per SD)	0.72*	0.58, 0.90
Parental Cognitions		
Self-efficacy (per SD)	0.62*	0.49, 0.78
Attitudes (per SD)	1.48*	1.12, 1.91
Descriptive Norms (per SD)	2.78*	2.21, 3.51
Parental Behavior		
Parental Screen Time (per SD)	1.76*	1.43, 2.17
Physical Environment Factors		
Television in Bedroom		
No	1.00	
Yes	1.40*	1.16, 1.70
Video Games in Bedroom		
No	1.00	
Yes	2.33	0.92, 5.95

Odds ratios for continuous and ordinal variables are expressed per each standard deviation change.

* *P* ≤ 0.05.

The results of the sensitivity analyses revealed that the significant predictors of screen time were the same regardless if imputed or best response values were used for parental attitudes and barriers among the 47 participants that did not engage in screen time. However, there was a better model fit (i.e., higher R-square) when the best response values were used (data not shown).

## Discussion

This study used the Ecologic Model of Sedentary Behavior [14] as a guide to examine associations between factors within the home setting and screen time among pre-school children. Several intrapersonal, interpersonal, and physical environment factors within the home setting were related to screen time, together explaining 64.2% of the variance in screen time. Parental cognitive variables explained most (37.9%) of this variance.

Consistent with the Ecologic Model of Sedentary Behavior [14], the findings of the present study suggest that there are multiple factors at different levels within a specific setting that simultaneously influence pre-school children’s screen time. To the authors knowledge, only one other study has used a similar approach in this age group [31]; however, many of the independent variables they examined were drawn from the physical activity literature and were not specific to screen time. Consequently, only two predictors (parental television rules, presence of play station) were identified. In contrast, by using screen time specific measures, the present study was able to identify a number of predictors, many modifiable, which collectively explained two thirds of the variance in screen time. Future research within representative samples of children is needed to determine whether these relationships are consistent at the population level.

While multiple levels of factors may influence pre-school children’s screen time, the findings of the present study suggest that parental cognitive factors may be of particular relevance. The observation that parental cognitions explained the most variance in screen time, particularly in 0- to 3-year-olds, is likely due to the control parents have over what young children are exposed to at home [6]. Thus, increasing parental awareness regarding the recommended levels of screen time, changing parental attitudes regarding children’s screen time engagement, as well as increasing parental self-efficacy to overcome barriers and say no to children’s request to engage in screen time may be potential strategies for future interventions. Although there is no consensus on the best approach for targeting parents [32], Golan’s and Wizman’s conceptual model may be promising [33]. While this model was developed for diet-related interventions, it encompasses many of the important factors identified in the present study regarding screen time [33]. For example, the first part of this model focuses on changing parents’ cognitions (e.g., self-efficacy, attitudes) by increasing parental knowledge and skills, which in turn leads to positive changes in parental modeling and positive changes within the home environment that ultimately facilitate healthy family habits [33]. Future research should evaluate interventions that target parents to determine the most appropriate approach. Furthermore, while this study focused on the home setting, future research should examine factors within other settings (e.g., child care centers, neighborhood environment) as outlined in the Ecologic Model of Sedentary Behavior [14].

Strengths of this study include the theoretical approach and the large sample size, which allowed for age-stratified analyses and the examination of multiple independent variables. A key study limitation is the cross-sectional design, which limits the ability to make causal inferences. In addition, since it is not feasible to obtain direct measurements of screen time in large population-based studies, the screen time measures were parental-reported. The information bias associated with these measures may have resulted in an underestimation of the true associations [34]. Furthermore, due to the low response rate and the fact that the main source of recruitment was licensed child care centers, which only 15% of pre-school children in the health region attend [35], the sample was not representative. Finally, while the questionnaire was pilot tested before distribution to the participants and all of the independent and dependent variables were adopted from national surveys or previous research, psychometric properties were not available for all of the variables.

## Conclusions

A large proportion of screen time in pre-school children was explained by intrapersonal, interpersonal, and physical environment factors within the home setting. Parental cognitive factors at the interpersonal level were of particular relevance. These findings suggest that future interventions aiming to foster appropriate screen time habits in pre-school children may be most effective if they target parents for behavioral change.

## Competing interests

The authors declare that they have no competing interests.

## Authors’ contribution

VC assisted with the design of the study, collected the data, led the statistical analysis, and wrote the initial draft of the manuscript. IJ assisted with the design of the study, provided insight and guidance on the statistical analysis, and revised the manuscript for important intellectual content. Both authors approve the version that has been submitted.

## Pre-publication history

The pre-publication history for this paper can be accessed here:

http://www.biomedcentral.com/1471-2458/12/539/prepub
